# Effect of 3D Printer Type and Use of Protection Gas during Post-Curing on Some Physical Properties of Soft Occlusal Splint Material

**DOI:** 10.3390/polym14214618

**Published:** 2022-10-31

**Authors:** Junichiro Wada, Kanae Wada, Mona Gibreel, Noriyuki Wakabayashi, Tsutomu Iwamoto, Pekka K. Vallittu, Lippo Lassila

**Affiliations:** 1 Department of Biomaterials Science, Turku Clinical Biomaterials Centre—TCBC, Institute of Dentistry, University of Turku, Itäinen Pitkäkatu 4B, 20520 Turku, Finland; 2 Department of Advanced Prosthodontics, Tokyo Medical and Dental University—TMDU, 1-5-45, Yushima, Bunkyo-ku, Tokyo 113-8510, Japan; 3 Department of Pediatric Dentistry/Special Needs Dentistry, Tokyo Medical and Dental University—TMDU, 1-5-45, Yushima, Bunkyo-ku, Tokyo 113-8510, Japan; 4 City of Turku Welfare Division, Oral Health Care, Lemminkäisenkatu 23, 20520 Turku, Finland

**Keywords:** 3D printing, degree of double bond conversion, mechanical property, microlayer structure, nitrogen gas, post-curing, soft occlusal splint

## Abstract

Despite the fact that three-dimensional (3D) printing is frequently used in the manufacturing of occlusal splints, the effects of the 3D printer type and post-curing methods are still unclear. The aim of this study was to investigate the effect of the printer type (digital light processing: DLP; and liquid crystal display: LCD) as well as the post-curing method with two different atmospheric conditions (air and nitrogen gas (N_2_)) on the mechanical and surface properties of 3D-printed soft-type occlusal splint material. The evaluated properties were flexural strength, flexural modulus, Vickers hardness (VHN), fracture toughness, degree of double bond conversion (DC%), water sorption, water solubility, and 3D microlayer structure. The printer type significantly affected all the evaluated properties. Flexural strength, flexural modulus, and fracture toughness were significantly higher when specimens were printed by a DLP printer, while VHN and DC% were significantly higher, and a smoother surface was noticeably obtained when printed by an LCD printer. The post-curing at an N_2_ atmosphere significantly enhanced all of the evaluated properties except water sorption, 3D microlayer structure, and fracture toughness. The current results suggested that the printer type and the post-curing methods would have an impact on the mechanical and surface properties of the evaluated material.

## 1. Introduction

Soft-type occlusal splints (SSs) are clinically used as aligners in orthodontic treatment [1] and as mouth guards to prevent sports-related injuries [2]. Meanwhile, soft and hard occlusal splints are widely applied in the treatment of temporomandibular disorders (TMDs) [3,4,5], the protection of prostheses and dental restorations [6,7], and the prevention of excessive attrition and/or occlusal trauma [8,9]. The clinical application of occlusal splints show no serious side effects, suggesting they are acceptable in terms of safety [3,8]. Previous studies revealed that hard-type occlusal splints (HSs) fabricated using heat-cured or auto-polymerizing polymethyl methacrylate (PMMA) would be preferred for the above-mentioned clinical applications rather than SSs [10,11,12,13]. However, several reports have indicated that SSs would be more effective in specific clinical aspects than HSs [4,14,15,16]. Halachmi et al. reported that the antagonists of HSs would be exposed to a higher risk of bending forces than those of SSs [14]. Seifeldin et al. revealed that the clinical application of SSs would result in earlier improvement of TMD symptoms than that of HSs [4]. In addition, Ariji et al. reported that HSs resulted in more widespread brain activity during jaw clenching than SSs [15], and Sriharsha et al. indicated that SSs would reduce patients’ stress levels during bruxism [16].

Conventionally, SSs are fabricated on a cast poured from an intraoral impression. The thermoplastic material is softened by heating and placed on the working cast. Using a vacuum or pressure-forming machine, the material closely adheres to the cast. SSs are more difficult to adjust their fitness and occlusal contact than HSs because thermoplastic materials become easily sticky with heat generation during cutting and hardly connect with PMMA, which is used to increase the thickness of SSs and/or improve their fitness. The above-mentioned difficulty in intraoral adjustments is one of the reasons why SSs are not widely applied for clinical situations compared to HSs. Furthermore, the dimensional changes of the elastic impression materials used for conventional impression taking and the dental stone used for working cast fabrication are potentially associated with a higher risk of inferior accuracy in the fitness of occlusal splints [17].

Recently, digital technology has played an important role in various dental fields. The three-dimensional (3D) printing method is rapidly becoming more common in dental appliance fabrications, including occlusal splints [18,19]. This method provides 3D objects built by a layer-by-layer replication with two-dimensional (2D) data acquired with computer-aided designing (CAD) and computer-aided manufacturing (CAM) systems [20,21,22,23]. The three-dimensional printing method can result in a higher fitting accuracy of the fabricated objects [24] because it includes fewer processing complications than conventional methods. In addition, 3D printing can constantly provide an accurate shape of the splints [20,21]. According to the above-mentioned advantages, 3D printing can result in a more predictable performance than the conventional methods [25]. Simultaneously, 3D printing is cost-effective and time-efficient because of the unnecessity of intermediate procedures and physical materials that are necessary for conventional methods [22,24].

Three-dimensional printing systems currently used in the dental field can be categorized into the following four types: (1) inkjet printing [26]; (2) extrusion printing [27]; (3) selective laser melting (SLM) [28]; and (4) stereolithography printing [29]. For occlusal splint fabrication, a stereolithography printing system is commonly used [20,30]. In this system, the photopolymer material is placed on the resin tray, and a light illumination is directly projected through the tray bottom to print the material. Digital light processing (DLP), in which the image is projected using a screen by reflecting the light, is the most common 3D printing system using stereolithography printing [31]. Although liquid crystal display (LCD), in which light comes from a light-emitting diode (LED) back panel and a mask is generated to block out the light, is also a commonly used system, dental materials developed for 3D printing methods are mainly based on using DLP 3D printers partially because the light intensity in DLP printers is typically higher than that in LCD printers [32,33]. On the other hand, a previous study indicated that both DLP and LCD printers provided clinically acceptable mechanical properties of 3D-printed interim prostheses [34]. Additionally, several studies suggested that the mechanical properties of 3D-printed objects would be affected by the 3D printer type [35,36]. However, it is still unclear how the printer type affects the mechanical properties of 3D printing materials used for SSs fabrication.

After the acquisition of 3D-printed objects, the unpolymerized resin should be removed using a solvent, followed by post-curing in a light polymerization chamber to finalize the polymerization [37,38]. The effect of the post-curing method on the mechanical properties of 3D-printed materials for denture base PMMA [39,40] and interim prostheses [41,42] has been previously investigated, suggesting that the mechanical properties of those materials would be affected by the post-curing method. Reymus et al. reported that the post-curing methods affected the degree of conversion of 3D-printed interim prostheses, and stroboscopic post-curing in a nitrogen gas (N_2_) atmosphere resulted in the highest degree of conversion [41]. Although a few studies have investigated the effect of the post-curing method on the mechanical properties of 3D-printed materials used for HSs [18,19], there is no report documenting the effect of post-curing on the mechanical properties of 3D-printed materials used for SSs.

Therefore, this study aimed to investigate the effect of 3D printer type (DLP and LCD) and post-curing atmosphere conditions (air and N_2_) on the mechanical and surface properties of 3D-printed material used for SSs. The evaluated mechanical properties were flexural strength, flexural modulus, surface hardness, water sorption, water solubility, and fracture toughness. Additionally, the degree of double bond conversion and geometry of the 3D microlayer structure, and microscopic surface condition were evaluated. The tested null hypotheses were as follows: (1) the printer type would have no impact on the evaluated properties of 3D-printed SSs material; and (2) the post-curing method would have no impact on the evaluated properties of 3D-printed SSs material.

## 2. Materials and Methods

### 2.1. Specimen Fabrication

**Figure 1:**
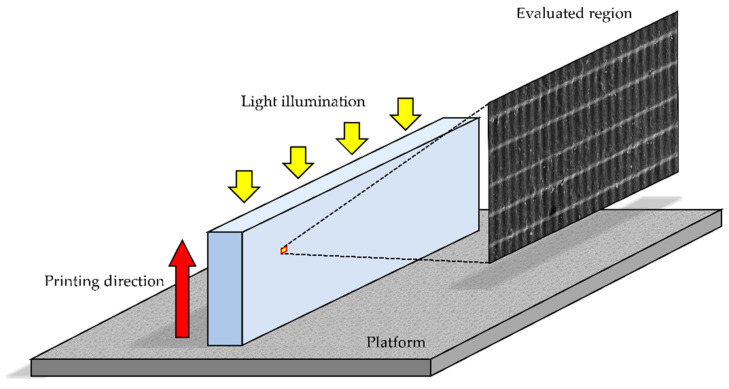
The printing direction (red arrow), direction of light illumination (yellow arrows) and evaluated region for Vickers hardness number (VHN) and three-dimensional (3D) microlayer structure.

**Figure 2:**
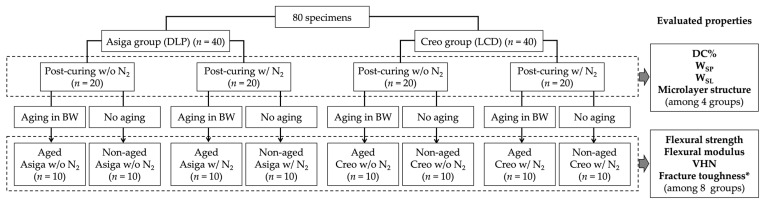
Flowchart of specimen fabrication. DLP: digital light processing; LCP: liquid crystal display; N_2_: nitrogen gas; BW: boiling water; DC%: degree of double bond conversion; WSP: water sorption; WSL: water solubility; and VHN: Vickers hardness number; Post-curing w/o N_2_: stroboscopic post-curing in the air atmosphere; Post-curing w/ N_2_: stroboscopic post-curing at a nitrogen gas atmosphere; *: For fracture toughness evaluation, additional 64 specimens were fabricated (*n* = 8/group).

Eighty bar-shaped specimens (3.0 mm × 10.0 mm × 60.0 mm) were 3D-printed using a photopolymerizing material for 3D-printed SSs (KeySplint^®^ Soft, Keystone Industries GmbH, Singen, Germany), which is based on methacrylate chemistries [43]. Half of them (*n* = 40) were printed by a DLP printer with a LED wavelength of 385 nm (Asiga MA^MT^, SCHEU-DENTAL GmbH, Iserlohn, Germany) (Asiga group), while the other half were printed by an LCD printer with a LED wavelength of 405 nm (Creo^TM^ C5, PLANMECA OY, Helsinki, Finland) (Creo group). Three-dimensional printing was performed in a horizontal direction with a layer thickness of 100 µm (Figure 1). The printed specimens were cleaned in 99% isopropanol for 10 min in an ultrasonic cleaning unit (Quantrex^®^ 90, L&R Ultrasonics, New Jersey, NJ, USA) to remove unpolymerized material, followed by stroboscopic post-curing on both sides using 2000 flashes with 10 Hz frequency at a wavelength of 300–700 nm and a pressure of 120 KPa (Otoflash G171, BEGO GmbH & Co, Bremen, Germany). For each group, half of the printed specimens (*n* = 20) were post-cured in the air atmosphere (without N_2_), while the other half were post-cured at an N_2_ atmosphere (with N_2_). Additionally, for each subgroup, half of the post-cured specimens (*n* = 10) were aged in boiling distilled water for 16 h, while the other half were not aged (Figure 2).

### 2.2. Flexural Strength and Modulus Testing

The flexural strength (MPa) and modulus (GPa) were assessed by conducting a 3-point bending test in the air atmosphere at room temperature for the printed specimens (*n* = 10/subgroup) by a universal testing machine (Model LRX; Lloyds Instruments Ltd, Hampshire, UK) using a load cell with a capacity of 2500 N. The crosshead speed was 5.0 mm/min and the distance between the supports of the test specimens was 50 mm. Each test was set to be finished when the specimen deflection reached 12 mm or the load reduction reached 10% of the maximum load.

### 2.3. Vickers Hardness (VHN)

As a measure of the surface hardness, Vickers hardness number (VHN) was measured. Two specimens were randomly selected from the specimens fabricated in 2.1., followed by the measurement of VHN at 10 different regions on each selected specimen using a Vickers hardness testing device (Duramin-5, Struers, Ballerup, Denmark) (*n* = 20/subgroup). A force of 9.81 N was applied for 5 s. Using a square-based pyramid diamond indenter, an indentation was created on each region. The diagonals of pyramidal indentation were measured with a microscopic scale of the testing device, and VHN was calculated using the following equation:

H = 0.1891 × F/d^2^ (1)

where H is VHN; F is the indenting force in newtons (N); and d is the mean length of two diagonals of the squared base of indentation in millimeters (mm).

### 2.4. Fracture Toughness (K_IC_)

For fracture toughness testing, sixty-four 3D-printed bar-shaped specimens (8.0 mm × 4.0 mm × 40.0 mm) were additionally fabricated and divided in the same manner as described in 2.1. (*n* = 8/subgroup). A single-edge notched bend (SENB) test was performed to measure fracture toughness. Each specimen was centrally notched with a depth of 3.0 mm using a double-sided diamond disk with 0.15 mm thickness (Komet, Brassler, Legmo, Germany). The depth of each specimen was checked to be standardized using a light microscope (Leica; Leica Microsystem GmbH, Wetzlar, Germany). Afterward, the notch was polished and sharpened by a straight-edged razor blade. A 3-point bending test was performed for each notched specimen using a universal testing machine. The crosshead speed was 1.0 mm/min and the distance between the supports of the test specimens was 32 mm. After testing, the notch length on the fracture surface of each specimen was measured 3 times using a light microscope, and the mean value of the 3 measurements was defined as the crack length in millimeters (mm). The fracture toughness was calculated in MPa m^1/2^ using the following equation:

K_IC_ = f(x)[(PL)/(BW^3/2^)]√10^−3^ (2)

and

f(x) = 3 (a/W)^1/2^{1.99 − (a/W)(1 − a/W)(2.15 − 3.93(a/W) + 2.7(a/W)^2^)}/{2[1 + 2(a/W)](1 − a/W)^3/2^}, (3)

where K_IC_ is the fracture toughness (MPa m^1/2^); P is the maximum load in newtons (N); L is the span distance in millimeters (32.0 mm); B is the specimen width in millimeters (mm); W is the specimen thickness in millimeters (mm); and a is the crack length in millimeters (mm).

### 2.5. Degree of Double Bond Conversion (DC%)

A Fourier-Transform Infrared (FT-IR) spectrometer (Frontier FT-IR spectrometer, PerkinElmer, Llantrisant, UK) was used for measuring the degree of double bond conversion (DC%) [44]. Measurements were conducted for randomly selected non-aged specimens (*n* = 5/subgroup). The ratio of the intensity of C=C double bond (aliphatic) absorbance peak at 1638 cm^−1^ was detected based on the aromatic reference peak at 1600 cm^−1^. As the control, the ratio in the unpolymerized material also was detected in the same manner. The DC% was calculated as a percentage (%) using the following equation:

DC% = [1 − (C_ali_/C_aro_)/(U_ali_/U_aro_)] × 100 (4)

where C_ali_ is the ratio of the aliphatic peaks of the polymerized specimen; C_aro_ is the ratio of the aromatic peaks of the polymerized specimen; U_ali_ is the ratio of the aliphatic peaks of the unpolymerized material; and U_aro_ is the ratio of the aromatic peaks of the unpolymerized material.

### 2.6. Water Sorption (W_SP_) and Solubility (W_SL_)

Water sorption (W_SP_) and solubility (W_SL_) assessment was performed for the same specimens used for flexural strength evaluation (*n* = 8/subgroup). First, the specimens were dried in a vacuum desiccator containing freshly dried silica at 37 ± 1 °C for 22 h and then at 23 ± 1 °C for 2 h (the first drying procedure). The initial weight (WT_1_) of each specimen was measured using a digital analytical balance (XS105; Mettler Toledo, Greifensee, Switzerland) to an accuracy of 0.1 mg. Then, the first drying procedure was continued until the weight decrease was less than 0.1 mg for all the specimens. Second, after stabilizing the weight of each specimen, the specimens were immersed in 50.0 mL of distilled water and stored at 37 °C for 30 days (the water immersion procedure). At 1, 2, 3, 7, 14, 21, 28, and 30 days after immersion into the water, the weight of water-immersed specimens was measured 60 s after being removed from the water and carefully dried with an absorbent paper (the weight at 30 days was named as WT_2_). Finally, in accordance with the same manner as the first drying procedure, the second drying procedure was continued until a stable weight (WT_3_) of each specimen was acquired. W_SP_ and W_SL_ were calculated in percentages (%) using the following equations [19,45,46]:

W_SP_ = [(WT_2_ − WT_3_)/WT_1_] × 100 (5)

and

W_SL_ = [(WT_1_ − WT_3_)/WT_1_] × 100 (6)

where WT_1_ is the initial weight of the specimen after storing it in a desiccator for 24 h in milligrams (mg); WT_2_ is the weight of the specimen after water immersion for 30 days in milligrams (mg); and WT_3_ is the stable weight of the specimen after the second drying cycle in milligrams (mg).

### 2.7. Three-Dimensional Microlayer Structure and Surface Condition

**Figure 3:**
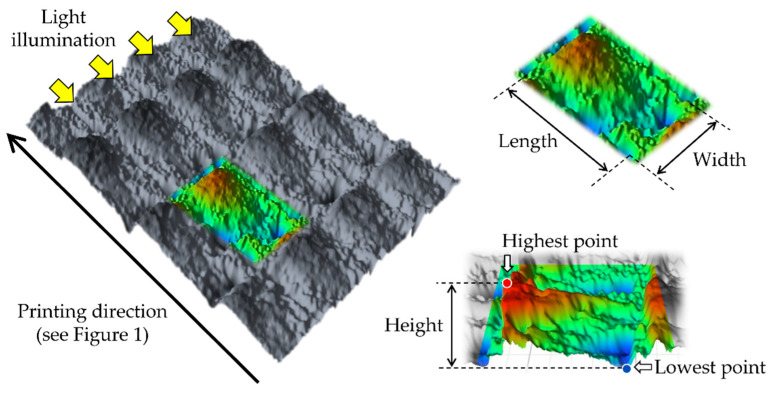
The measures for the size of the 3D microlayer structural unit.

One specimen was randomly selected from each non-aged subgroup and gold-sputtered for the measurement of the size of the 3D microlayer structural unit (width, length, and height) in micrometers (µm) (Figure 3). The measurement was performed in 10 different surface regions (236 µm × 314 µm) on each selected specimen using a 3D optical profilometer (OP) (ContourGT-I, Bruker Nano, Inc., Tucson, AZ, USA). In addition, the surface condition of the selected specimen was optically observed using a scanning electron microscope (SEM) (JSM-5500, JEOL Ltd., Tokyo, Japan).

### 2.8. Statistical Analysis

For all acquired data, the equality of variance was validated by the Levene test. As shown in Figure 2, the flexural strength, flexural modulus, VHN, and fracture toughness were dependent on three variables (the printer type, post-curing methods, and the aging procedure), while the DC%, W_SP_, W_SL_, and size of 3D microlayer structure were dependent on two variables (the printer type and post-curing methods). Therefore, a 3-way analysis of variance (ANOVA) was performed to detect the effect of the printer type, post-curing methods, and aging in boiling water as the independent variables on the flexural strength, flexural modulus, VHN, and fracture toughness. Meanwhile, for the DC%, W_SP_, W_SL_, and size of 3D microlayer structure, the effect of the printer type and post-curing methods as the independent variables was detected using a 2-way ANOVA. Additionally, all the evaluated properties were statistically compared among all subgroups with a 1-way ANOVA followed by a Tukey multiple comparison post hoc analysis. The statistical software (IBM SPSS Statistics v28.0, IBM, Redmond, WA, USA) was used for all statistical analyses. The significance level was set at 5% (*α* = 0.05).

## 3. Results

The *p*-values acquired by multiple-way ANOVA are shown in Table 1. The printer type and aging in boiling water significantly affected all the evaluated properties (*p* < 0.05). On the other hand, the post-curing method did not significantly affect the fracture toughness, W_SP_, and size of the 3D microlayer structural unit.

The mean values and standard deviations of the flexural strength, flexural modulus, VHN, and fracture toughness are presented in Table 2. The evaluated properties after aging in boiling water generally showed inferior values when compared to those without the aging. Regardless of the post-curing method and aging in boiling water, the printer type significantly affected all the evaluated properties (*p* < 0.001 for all the properties other than the flexural strength (*p* = 0.039) and flexural modulus (*p* = 0.003) in the non-aged groups post-cured with N_2_) except for the flexural modulus in the non-aged groups post-cured without N_2_ (*p* = 0.300). The post-curing at an N_2_ atmosphere significantly enhanced the flexural strength, flexural modulus, and VHN of non-aged Asiga (*p* < 0.001, *p* < 0.001, and *p* < 0.001, respectively), aged Asiga (*p* = 0.010, *p* = 0.009, and *p* < 0.001, respectively), non-aged Creo (*p* < 0.001, *p* = 0.007, and *p* < 0.001, respectively) specimens. However, its effect on the flexural strength, flexural modulus of aged Creo specimens was non-significant (*p* = 1.000 and *p* = 1.000, respectively). Furthermore, the post-curing method did not significantly affect the fracture toughness of neither aged and non-aged Asiga subgroups (*p* = 0.968 and *p* = 0.902, respectively) nor aged and non-aged Creo subgroups (*p* = 0.146 and *p* = 0.968, respectively).

**Figure 4:**
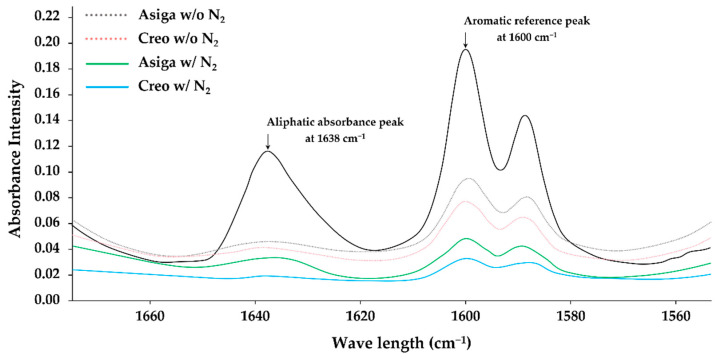
The typical IR spectra of the aliphatic absorbance peak and the aromatic reference peak used for DC% calculation.

The mean values and standard deviations of the DC%, W_SP_, W_SL_, and size of the 3D microlayer structure unit are shown in Table 3. Additionally, the typical IR spectra of the peaks used for the DC% calculation are shown in Figure 4. Asiga groups displayed higher W_SP_ than the Creo groups regardless of the post-curing method (*p* < 0.001 and *p* < 0.001 for groups post-cured with and without N_2_, respectively), while the post-curing method showed no significant effect on the W_SP_ (*p* = 0.930 and *p* = 0.966 for Asiga and Creo groups, respectively). For the W_SL_, the post-curing method significantly affected the W_SL_ (*p* < 0.001 and *p* < 0.001 for Asiga and Creo groups, respectively). Additionally, Asiga group post-cured with N_2_ displayed the lowest W_SL_ value. Regardless of the post-curing method, the Creo groups displayed a significantly smaller size of the 3D microlayer structure unit (*p* < 0.001, *p* < 0.001, and *p* < 0.001 for the width, length, and height, respectively), while the difference between subgroups post-cured with and without N_2_ was not significant within the same printer group (*p* = 0.994 and *p* = 0.999 for the width, *p* = 0.960 and *p* = 0.989 for the length, and *p* = 0.827 and *p* = 0.998 for the height of Asiga and Creo subgroups, respectively). The printer type showed no significant effect on the DC% (*p* = 0.365 and *p* = 0.171 for groups post-cured without and with N_2_, respectively), while the post-curing method significantly affected the DC% in both groups (*p* = 0.034 and *p* = 0.012 for Asiga and Creo groups, respectively).

**Figure 5:**
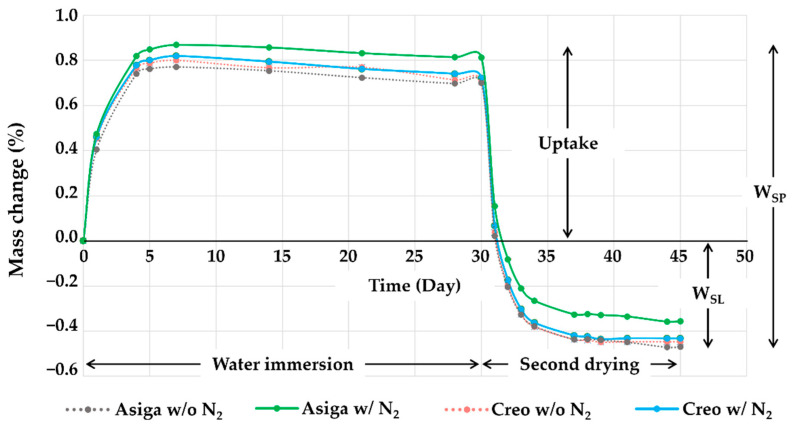
The representative plots of mass changes (%) against time during the water immersion and the second drying procedures. It was noted that saturation was achieved 7 days after water immersion and drying was completed 15 days after drying began in all groups.

**Figure 6:**
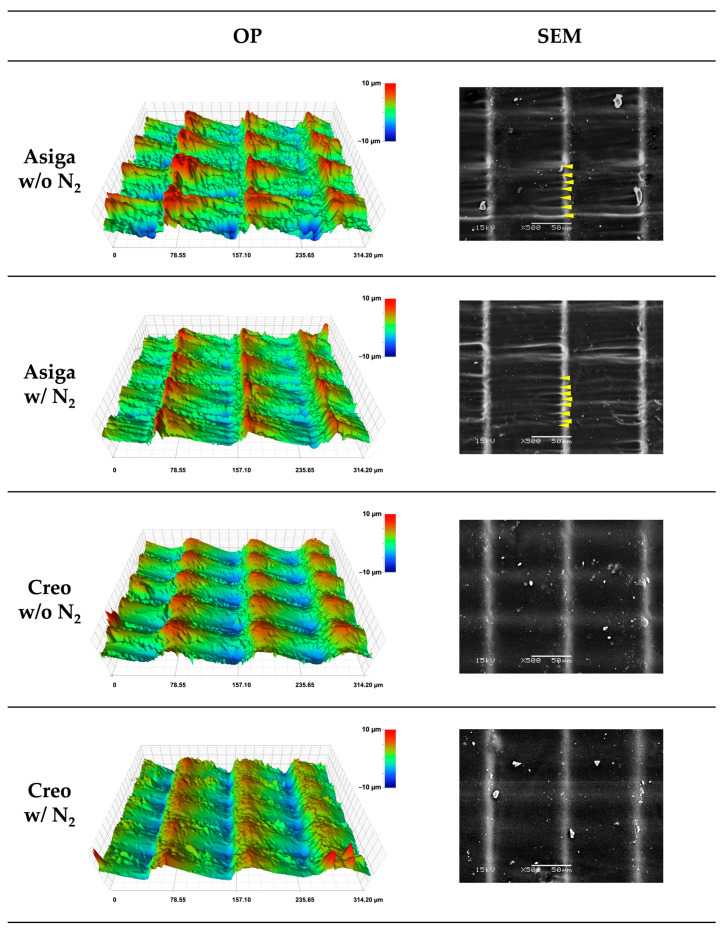
Optical profilometer (OP) images and scanning electron microscope (SEM) images (×400 at 15 kV) of microlayer structure in typical specimens. It was noted that the greater and rougher microlayer structural units with wavelike structures (yellow arrows) were observed in Asiga specimens compared to Creo specimens and no obvious difference was found between specimens post-cured with and without N_2_.

**Figure 7:**
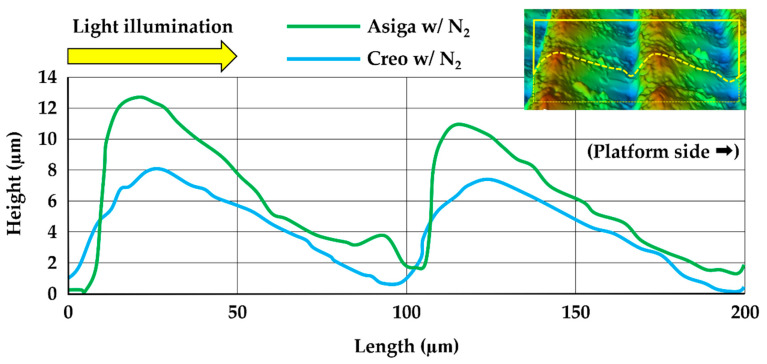
The typical cross-sectional profiles of the specimens post-cured with N_2_ obtained by Optical profilometer (OP). A significantly increased surface height variation was noted in Asiga specimen.

The representative plots of mass changes in time (%) during the water sorption and solubility test are shown in Figure 5. The saturation was reached 7 days after water immersion in all groups. During the second dying procedure, all groups were completely dried up 15 days after drying began. The typical images obtained by the OP and SEM are shown in Figure 6. Additionally, the typical cross-sectional profiles which were vertical to the printing direction are shown in Figure 7. The OP images revealed the greater and rougher microlayer structures in Asiga groups compared to Creo groups. The SEM images as well as the OP images revealed that the rougher surface with wavelike structures was obtained in Asiga groups compared to the Creo groups regardless of the post-curing method. However, no obvious difference was found between specimens post-cured with and without N_2_ in both Asiga and Creo groups.

## 4. Discussion

In this study, the effect of the printer type and post-curing conditions on the mechanical properties, including the flexural strength, flexural modulus, surface microhardness (VHN), fracture toughness, water sorption (W_SP_), and water solubility (W_SL_) was demonstrated in a 3D-printed soft-type occlusal splint (SS) material. In addition, the degree of double bond conversion (DC%) and size of the 3D microlayer structure units were also evaluated. The total results revealed that the printer type significantly affected the mechanical properties, DC%, and the size of the 3D microlayer structure unit of the 3D-printed material used for SSs, and that the post-curing at an N_2_ atmosphere enhanced the mechanical properties and DC% of the 3D-printed materials. Therefore, the two null hypotheses were rejected.

Regarding the printer type, the flexural strength, flexural modulus, and fracture toughness in Asiga groups were significantly higher than those in Creo groups. It might be due to the light intensity of the DLP printing system (Asiga printer), which is basically higher than that of the LCD printing system (Creo printer) [31]. In this study, although two different 3D printers with different printing systems (DLP and LCD) were used, all the specimens were printed in accordance with the manufacturer’s guidelines in which DLP printers were recommended to be used. Using different recipes for the printing and/or post-curing might enhance the mechanical properties of the specimens printed by Creo printer [34]. On the other hand, the VHN and DC% in Asiga groups were significantly lower than those in Creo groups, suggesting that a higher DC% could lead to a higher VHN. Additionally, the printer type significantly affected the size of the 3D microlayer structural unit. Because the printing layer thickness was unified (100 µm), the difference in the length of the microlayer structural unit between Asiga and Creo groups was smaller when compared to that in the width and height. In contrast, the height of the microlayer structural unit in Asiga group (10.5 µm) was dramatically higher than that in Creo group (6.0 µm), suggesting that Creo groups showed a smoother surface than Asiga groups (Figure 6 and Figure 7). Asiga printer had a higher light intensity compared to Creo printer, leading to a larger polymerized area. Furthermore, the deviation of the height in Asiga groups was greater than that in Creo groups. For the DLP printing system used in Asiga printer, it would be impossible to homogenize the light intensity to print the objects since the integration pattern of the projector is not fixed at each layer [47]. The heterogeneity with respect to the light intensity might lead a greater deviation in the height of the microlayer structural unit in Asiga group. Our findings also indicated that the Creo printer could homogenize the quality of 3D-printed objects more than the Asiga printer even if some of the evaluated mechanical properties in the Creo groups showed lower values.

In this study, aged specimens showed critically lower flexural strength, flexural modulus, VHN, and fracture toughness than non-aged ones. The aging method used in this study (immersion in boiling distilled water for 16 h) was a common way to evaluate the effect of hydrolytic and thermal breakdown in dental materials [48]. This aging method is efficient to evaluate the material stability against deterioration [49]. It is interesting to note that the flexural strength and modulus in aged Asiga groups were significantly enhanced by the post-curing with N_2_ (improvement of 2.3 MPa in the flexural strength and improvement of 0.41 Gpa in the flexural strength), while the post-curing with N_2_ did not affect those mechanical properties in aged Creo groups (Table 2). Additionally, there was no significant difference in the VHN between aged Asiga and Creo groups when post-cured with N_2_, while the VHN in aged Asiga groups was significantly lower than in aged Creo groups post-cured without N_2_. These findings suggested that the post-curing with N_2_ could dramatically enhance the material stability against deterioration of the specimens printed by Asiga printer compared to those printed by Creo printer. Further studies should be required to clarify the effect of the printer type and post-curing methods on the resistant character of 3D-printed SSs against long-term usage in the clinical situation.

The post-curing is needed for the 3D-printed objects to finalize the polymerization after the printing process. It has been reported that the post-curing methods would affect the DC% [40,41,50,51]. In this study, the post-curing with N_2_ significantly enhanced the DC% both in Asiga (an improvement of 9.2%) and Creo groups (an improvement of 10.8%) (Table 3). The existence of an N_2_ can reduce the oxygen inhibition layer [52], leading to a higher DC% [40,41]. The higher DC% of the specimens post-cured with N_2_ might explain the higher flexural strength (an improvement of 3.0 MPa and 3.8 MPa in Asiga and Creo groups, respectively), flexural modulus (an improvement of 0.21 GPa and 0.09 GPa in Asiga and Creo groups, respectively), VHN (an improvement of 0.7 and 0.37 in Asiga and Creo groups, respectively), and water solubility (an improvement of 0.114% and 0.015% in Asiga and Creo groups, respectively) when compared to the specimens post-cured without N_2_. These findings suggested that the post-curing with N_2_ would be more effective when using the Asiga printer than when using the Creo printer, except for the flexural strength. The post-curing time is maintained regardless of using N_2_. Furthermore, using N_2_ during the post-curing is basically more cost-effective than using other gases such as an argon atmosphere. Therefore, our findings suggested that post-curing with N_2_ would be a time-efficient and cost-effective method to improve the mechanical properties of 3D-printed SSs. However, although a numerical improvement of 10–20% was revealed in those mechanical properties, the clinical impacts of their improvements are still unclear. A further clinical study is needed to evaluate how the improvement in mechanical properties by the post-curing with N_2_ will impact the performance of 3D-printed SSs in the actual oral cavities. On the other hand, our findings revealed that the post-curing method did not significantly affect fracture toughness and water sorption. The SENB test used to evaluate the fracture toughness in this study has been known to give results mainly affected by the inner structure of the specimen [53]. The limited effect of the existence of an N_2_ during the post-curing, which can enhance the polymerization only on the surface of specimens, may help to explain the invalidity of the post-curing method on the fracture toughness. Arima et al. suggested that water sorption could be mainly affected by the chemical characteristics of polymers against that of water molecules, while water solubility could be mainly affected by the degree of polymerization of the polymer [54]. Although the chemical composition of the evaluated material was not provided by the manufacturer, Arima’s suggestion might help to explain the significant effect of the post-curing condition on the water solubility noticed in this study. Additionally, it should be noted that the changing ratio of weight during the water immersion procedure started decreasing a little in a week (Figure 5), suggesting the possibility of some degradation change within the specimens. 

This study had several limitations. First, the DC% was measured only on the surface of specimens. Although the DC% of the inside of the specimens was unclear, it might affect the evaluated properties. Second, the testing conditions including the shape of specimens and aging conditions critically differed from the clinical situations. Therefore, further clinical trials are needed to evaluate the clinical performance of the 3D-printed SSs. Additionally, the wear resistance of 3D-printed SSs should also be considered for future studies. Third, there is no available information on the mechanical properties of traditional SSs. Therefore, it is still unclear if the mechanical properties shown in this study would be enough for clinical application. Finally, the exact chemical composition of the evaluated material has not been provided by the manufacturer, nor in the previous studies. Due to the absence of the information, it is still unclear if our findings can be generalized to other materials for 3D-printed SSs. Additionally, a chemical composition analysis is needed to be performed in further studies to clarify the relationship between the change in mechanical properties of the evaluated material and their chemical composition.

## 5. Conclusions

Within the limitations of this study, it can be concluded that the printer type and post-curing conditions have an impact on the mechanical and surface properties of 3D-printed soft-type occlusal splint material. A digital light processing (DLP) printer can provide higher flexural strength, flexural modulus, and fracture toughness of the evaluated 3D-printed SSs material, while a liquid crystal display (LCD) printer can provide a smoother surface with a higher degree of conversion, lower water sorption, and Vickers hardness. Additionally, the post-curing at a nitrogen gas atmosphere can numerically improve its mechanical properties (DC%, flexural strength, flexural modulus, VHN, and water solubility) by 10–20%, especially when using a DLP printer.

## Figures and Tables

**Table 1:** *p*-values acquired by 3- and 2-way ANOVA for the evaluated mechanical properties.

Variable	Flexural Strength ^a^	Flexural Modulus ^a^	Vickers Hardness Number (VHN) ^a^	Fracture Toughness ^a^	Degree of Double Bond Conversion (DC%) ^b^	Water Sorption (W_SP_) ^b^	Water Solubility (W_SL_) ^b^	Size of Three-Dimensional (3D) Microlayer Structural Unit ^b^		
								Width	Length	Height
Printer type	<0.001 *	<0.001 *	<0.001 *	<0.001 *	0.015 *	<0.001 *	<0.001 *	<0.001 *	<0.001 *	<0.001 *
Post-curing method	<0.001 *	<0.001 *	<0.001 *	0.484	<0.001 *	0.457	<0.001 *	0.922	0.898	0.638
Aging in boiling water	<0.001 *	<0.001 *	<0.001 *	<0.001 *	-	-	-	-	-	-

^a^: *p*-values acquired by 3-way ANOVA; ^b^: *p*-values acquired by 2-way ANOVA; *: *p* < 0.05 is significant.

**Table 2:** Mean values and standard deviations of flexural strength, flexural modulus, VHN, and fracture toughness, and results of 1-way ANOVA statistical analysis.

Printer Type	Post- Curing	Aging in BW	Flexural Strength (MPa)		Flexural Modulus (GPa)		VHN		Fracture Toughness (MPa m^1/2^)	
				_#_		_#_		_#_		_#_
Asiga	w/o N_2_	−	31.1 ± 1.5	^a^	0.73 ± 0.06	^ab^	5.15 ± 0.25	^a^	2.66 ± 0.10	^a^
		+	22.1 ± 1.0	^b^	0.48 ± 0.03	^c^	4.28 ± 0.26	^b^	1.49 ± 0.11	^b^
	w/N_2_	−	34.1 ± 1.4	^c^	0.94 ± 0.03	^d^	5.85 ± 0.25	^c^	2.60 ± 0.11	^a^
		+	24.4 ± 0.9	^d^	0.89 ± 0.03	^e^	5.19 ± 0.23	^a^	1.44 ± 0.05	^b^
Creo	w/o N_2_	−	28.3 ± 1.9	^e^	0.68 ± 0.07	^a^	5.89 ± 0.29	^c^	2.03 ± 0.10	^c^
		+	18.8 ± 1.2	^f^	0.37 ± 0.06	^f^	4.90 ± 0.28	^d^	1.05 ± 0.13	^d^
	w/N_2_	−	32.1 ± 1.4	^a^	0.77 ± 0.06	^b^	6.26 ± 0.24	^e^	2.08 ± 0.07	^c^
		+	18.6 ± 1.7	^f^	0.36 ± 0.06	^f^	5.19 ± 0.25	^a^	1.18 ± 0.08	^d^

VHN: Vickers hardness number; and BW: boiling water; post-curing w/o N_2_: stroboscopic post-curing in the air atmosphere; post-curing w/N_2_: stroboscopic post-curing at a nitrogen gas atmosphere; −: without aging (non-aged group); and +: with aging (aged group); ^#^ the same superscripted letters indicate groups not statistically significantly different when compared by 1-way ANOVA and post hoc analysis with Tukey multiple comparisons.

**Table 3:** Mean values and standard deviations of DC%, size of 3D microlayer structural unit, water sorption, and water solubility, and results of 1-way ANOVA statistical analysis.

Printer Type	Post- Curing	DC% (%)		W_SP_ (%)		W_SL_ (%)		Size of 3D Microlayer Structural Unit					
								Width (μm)		Length (μm)		Height (μm)	
			_#_		_#_		_#_		_#_		_#_		_#_
Asiga	w/o N_2_	75.7 ± 4.3	^a^	1.174 ± 0.004	^a^	0.470 ± 0.004	^a^	61.2 ± 0.5	^a^	101.3 ± 0.5	^a^	10.5 ± 1.3	^a^
	w/N_2_	84.9 ± 5.9	^bc^	1.173 ± 0.004	^a^	0.356 ± 0.024	^b^	61.3 ± 0.5	^a^	101.2 ± 1.0	^a^	10.2 ± 0.7	^a^
Creo	w/o N_2_	80.8 ± 3.7	^ab^	1.159 ± 0.008	^b^	0.447 ± 0.011	^c^	51.3 ± 0.4	^b^	97.5 ± 0.5	^b^	6.0 ± 0.4	^b^
	w/N_2_	91.6 ± 5.1	^c^	1.158 ± 0.004	^b^	0.432 ± 0.017	^d^	51.3 ± 0.5	^b^	97.6 ± 0.4	^b^	6.1 ± 0.2	^b^

DC%: degree of double bond conversion; W_SP_: water sorption; and W_SL_: water solubility; post-curing w/o N_2_: stroboscopic post-curing in the air atmosphere; post-curing w/N_2_: stroboscopic post-curing at a nitrogen gas atmosphere; ^#^ the same superscripted letters indicate groups not statistically significantly different when compared by Tukey multiple comparisons post hoc analysis (*p* > 0.05).

## Data Availability

The data presented in this study are available on reasonable request from the corresponding author.
